# Racing Clean in a Tainted World: A Qualitative Exploration of the Experiences and Views of Clean British Elite Distance Runners on Doping and Anti-Doping

**DOI:** 10.3389/fpsyg.2021.673087

**Published:** 2021-07-08

**Authors:** Jake Shelley, Sam N. Thrower, Andrea Petróczi

**Affiliations:** ^1^School of Life Sciences, Engineering and Computing, Faculty of Science, Engineering and Computing, Kingston University, London, United Kingdom; ^2^Department of Life Sciences, University of Roehampton, London, United Kingdom

**Keywords:** doping, clean sport, distance running, elite sport, performance enhancement, anti-doping legitimacy, clean athletics

## Abstract

**Background:** Doping has been a prominent issue for the sport of athletics in recent years. The endurance disciplines, which currently account for 56% of the global anti-doping rule violations in athletics, appear to be particularly high risk for doping.

**Objective:** Using this high-risk, high-pressure context, the main purpose of this study was to investigate the human impact of doping and anti-doping on “clean” athletes. The secondary aim of the study was to better understand the reasons for, and barriers to, competing “clean” among this group of athletes.

**Method:** Semi-structured interviews were conducted with 11 elite distance runners from the UK to explore: (1) the reasons and motivations for competing clean. (2) Perceptions of the anti-doping system, and experiences of being part of that system. (3) Views on the prevalence and causes of doping and the impact of doping on the lives of clean athletes. The interviews were audio-recorded, transcribed verbatim, and analysed using Reflexive Thematic Analysis.

**Results:** Four major themes were identified: (1) The participants in this study have not been tempted to use prohibited substances or methods; they compete in their sport for the personal satisfaction of seeing how good they can be, rather than in pursuit of winning at all costs. (2) Anti-doping does not currently prevent doping effectively and is not implemented evenly across the globe. (3) Doping was perceived as a major issue and was felt to be borne out of certain sporting cultures in which doping is enabled. (4) Doping has impacted the careers of clean athletes in irreversible ways and presents a continuing challenge to the psychological preparation for competition.

**Conclusions:** Clean athletes suffer negative consequences from both doping and anti-doping. ADOs must collaborate across borders to ensure a more even implementation of anti-doping activities, to facilitate a more level playing field on the global stage. ADOs must also acknowledge the existence of a large group of athletes who would never consider deliberately doping and make anti-doping work for these athletes too.

## Introduction

Athletics has been linked to doping more than any other sport except cycling. The doping problem in athletics, and in the middle- and long-distance events particularly, is clearly demonstrated in the latest statistics from the Athletics Integrity Unit (AIU). Of the 465 athletes currently suspended from competing in athletics worldwide, 258 (55.5%) are middle- or long-distance runners (Athletics Integrity Unit, 2021). In the past three summer Olympic Games (2008, 2012, 2016), 108 medals were awarded in the endurance track events (800 m—marathon) and 15 of those medals (13.9 %) were won by athletes who have at one time served a doping suspension. A further 18 of the medals (16.7 %) were won by athletes who have been personally coached by someone who has been charged with doping offences. Another 60 medals (55.6%) were won by athletes from countries whose National Anti-Doping Organisation (NADO) has been declared non-compliant or been placed on the watch list, or whose Doping Control laboratory has had its accreditation suspended by the World Anti-Doping Agency (WADA) at one point. These figures indicate that significant levels of doping are present at the highest level of endurance athletics, which creates a vicious circle. Both the true and perceived prevalence of doping make athletics “high risk” for doping.

### Environment: Reasons for Distance-Running Being a High-Risk Sport for Doping

Along with the significant existing levels of doping, there are two further features of distance running which makes the sport “high risk” for doping: (1) the way performance is measured and (2) the financial gain attached to doing well in the sport. Sports such as athletics, weightlifting and cycling, where performance is measured in physical units, like centimetres, grammes, or seconds, are generally considered to be the most prone to doping (Pitsch et al., 2007). The nature of these sports means that the results are directly correlated to a certain physical attribute, whether that be speed, strength, or endurance, and it follows that they would be the sports where the enhancement of one or more of those attributes through doping can have the biggest influence on results. The total amount of money available in a sport is another factor thought to contribute to doping prevalence (Frenger et al., 2012; Westmattelmann et al., 2019).

In terms of financial incentives, there has been an influx of prize money in the road racing scene over the past 20 years. The total prize money awarded in road races increased from just under $11 million in 2000, to its current level of ~$24 million where it has remained stable for the past decade (Association of Road Running Statisticians, 2017). Furthermore, a large proportion of the athletes vying for the appearance money and prize money at the major road races around the world come from either Kenya or Ethiopia where the 2019 GDP per capita (USD) was $1816 and $855, respectively (The World Bank, 2020). It is not only the race winners who can do well financially in distance running; some high-profile athletes may earn up to $100,000 or more simply for appearing in a major marathon (Scott-Elliot, 2013), while other races offer thousands of dollars to each of the first 10 finishers (Gault, 2019).

### Factors Influencing Athletes' Decisions About Doping and Competing Clean

Several theories have been proposed to explain the fundamental determinants of behavioural choices. One of the most prominent models is the Theory of Planned Behaviour (Ajzen, 1991) which states that attitudes, social norms, and perceived behavioural control together make up the factors contributing to an individual's intentions, and an individual's intentions predict their actions. Focusing on the decision-making process that leads to a behaviour choice, the Behavioural Reasoning Theory (BRT) (Westaby, 2005) goes one step further by stating that not only do global motives (attitudes, social norms, perceived control) predict intentions (which in turn predict behaviour), but also that reasons predict intentions. Reasons often become stronger after the behaviour is executed through post-decision rationalisation processes (Westaby, 2005). Reasons are therefore useful vehicles for retrospective exploration of motives and decisions about doping or clean sport behaviour (Petróczi et al., 2017). Furthermore, doing something and not doing it rely on separate goals that thus have their own distinct set of reasons (Richetin et al., 2011), as has also been evidenced in relation to choices about doping (Overbye et al., 2013). It is therefore not possible to explain an athlete's decision to compete clean by exploring another group of athletes' reasons for doping, and assuming that the reverse of those reasons explains the opposite choice. To understand choices about clean sport behaviour, one must explore the specific set of reasons for being and remaining clean.

Applying the BRT model to doping decisions, it may be that; (1) athletes' decisions about clean sport behaviour are influenced by the values and beliefs they hold about doping use, and their perceptions about the environment (perceived prevalence, legitimacy of anti-doping including effectiveness and fairness of testing); and (2) the dynamic between values, beliefs and perceived environment is aptly captured in the self-identified motives for avoiding doping.

#### Values, Beliefs, and Attitudes

A 2014 meta-analysis collated the evidence on the predicting factors of doping intentions and behaviours (Ntoumanis et al., 2014). The results showed that a positive attitude towards doping was one of the strongest positive correlates of doping intentions and behaviours, and that morality had the strongest negative correlation. A 2016 meta-analysis likewise assessed the predictors of doping intentions but focussed specifically on elite athletes (Blank et al., 2016). The results again pointed to attitudes as a significant predictor of doping intentions and behaviour.

Since the publication of those two reviews, further studies have investigated the relationship between an athlete's values and beliefs, and their doping intentions. A recent study (Mortimer et al., 2020) reported that athletes with a strong moral identity are less likely to use banned substances, and similar findings have also been reported in several other recent studies across various sporting populations (Boardley et al., 2014; Allen et al., 2015; Madigan et al., 2016; Mallia et al., 2016; Kavussanu and Ring, 2017).

#### Perceived Prevalence

The relatively high prevalence of doping in distance running may send a message to aspiring runners about what it takes to succeed at the top level. In the situation where athletes believe that doping is the norm in their sport, doping may become an accepted part of being an elite athlete, as appears to have been the case in cycling in the past (Lentillon-Kaestner, 2013; Marty et al., 2015). The aforementioned meta-analyses (Ntoumanis et al., 2014; Blank et al., 2016) also both identified social/subjective norms as positive correlates with doping intentions and behaviours. A more recent meta-synthesis of qualitative research provided further support to indicate that a perception that others are doping may encourage athletes to perform such behaviours (Williams et al., 2020).

#### Procedural Legitimacy: Perceptions of Anti-Doping Effectiveness and Fairness

Athletes' perceptions of the effectiveness and fairness of the anti-doping system has been widely studied, and the literature in that area was the subject of a recent mapping review (Woolway et al., 2020). It is noted in this review that the greater the extent to which an athlete views the anti-doping rules and regulations as legitimate, the more likely that athlete will be to comply with those rules and regulations. Perceived legitimacy can therefore have a direct impact on the success of anti-doping organisations in establishing doping-free sport. One of the key insights from the review was that, although the anti-doping rules and regulations are now globally harmonised, there is still a requirement for better global harmonisation of the *implementation* of those rules and that the athletes would perceive the anti-doping system to be fairer if they felt that the rules were being applied evenly across all countries. The review also highlighted the need for better communication between Anti-Doping Organisations (ADOs) and athletes, to highlight progress in detection methods and to provide greater transparency around testing selection. The review concluded by suggesting that greater support for clean athletes to manage their anti-doping requirements would further enhance the perceptions of anti-doping legitimacy (Woolway et al., 2020).

#### Athletes' Reasons for Competing Clean

Whilst there is a plethora of literature investigating reasons for doping (Ntoumanis et al., 2014; Blank et al., 2016; Williams et al., 2020), the literature specifically focussing on the drivers for clean sport behaviour is less abundant. The simple lack of a motive to dope is not automatically a motivator to compete clean (Petróczi et al., 2017). Prior studies looking into athletes' motives for competing clean have shown them to be diverse and complex. Although testing and education are the central strategies to reduce doping in sport (WADA, 2021a,b,c), these are not generally cited by athletes as their reasons for not doping in qualitative research (Williams et al., 2020). Personal ethical standards, anticipated feelings of shame and guilt, and the influence of significant others, are instead the main factors that athletes point to when questioned on why they do not used prohibited means (Bloodworth and McNamee, 2010; MacNamara and Collins, 2014). Motives for clean sport behaviour are driven by values and early childhood experiences, viewing doping as cheating and a threat to the sport they love, as well as valuing authenticity and long-term health (Overbye et al., 2013; Lazuras et al., 2017; Whitaker et al., 2017; Kegelaers et al., 2018; Petroczi et al., 2021).

### Aims

While the previous research is undoubtedly useful, the inclusion of athletes from a variety of sports, countries and developmental levels within single studies perhaps leads to a loss of detail within each particular group. The reasons for and against doping cannot be generalised across different sporting cultures because there will be different drivers within (Kirby et al., 2011). Competing within a sport that has been tarnished by doping undoubtedly has an effect on all the athletes, and not only those who have committed the offences. Losing out on medals, opportunities and sponsorship deals are one aspect that clean athletes deal with. Lost opportunities may not apply directly to all but altered personal goals about what they felt was achievable is present in clean athletes' lives (Petroczi et al., 2021).

Yet, understanding the ways in which clean athletes are impacted has rarely been studied. Clean athletes, being the non-problematic portion of the athlete population, have been undeservedly overlooked in the research quest to build an evidence base to inform anti-doping policies. This study will therefore focus within a single country (UK) and a single sport (distance running), at the elite level, to delve into the details of that specific sporting context and to explore the experiences of doping and anti-doping that are particular to that sporting sub-culture. The purpose of the current study, therefore, is to explore the experiences and views on doping and anti-doping doping among clean elite middle- and long-distance runners from the United Kingdom. This approach will enable a focussed perspective on the highly publicised and prevalent issue of doping within a unique sporting culture. By placing the spotlight on clean elite distance runners, the current study will provide an insight into the experience of being impacted by the doping of others, as well as presenting their views on being part of the doping control system. This seldom-studied area will be addressed through the following research questions:

RQ1– What are the reasons and motivations underpinning elite distance runners' decision to complete clean?
RQ2– How do elite distance runners perceive the effectiveness of the anti-doping system, and what has it been like to be part of that system?
RQ3– How do elite distance runners perceive the prevalence and causes of doping in their sport, and how has doping affected their lives and careers?

## Methods

Based on the exploratory nature of the research questions, a qualitative approach was used. Qualitative research is an approach for exploring and understanding the meaning individuals, or groups, ascribe to social or human problems (Creswell and Creswell, 2017). As such, this approach enabled the first author to have detailed and wide-ranging conversations with the participants and thereby examine in detail their experiences and views on doping and anti-doping.

This current study was conducted from an interpretivist philosophical position, which emphasises the understanding and meaning people (i.e., elite distance runners) create for, and attribute, to their experiences (Poucher et al., 2020). Interpretivism is based on a relativist ontology (i.e., multiple individual realities) and a subjectivist epistemology (i.e., knowledge is constructed and subjective; Poucher et al., 2020). As such, the authors have a role as co-constructors of knowledge, and in interpreting the meaning of the lived experiences shared during the interviews. The following sub-sections outline how decisions made throughout the study are consistent with the assumptions that underpin interpretivism.

### Participants

In order to address the research questions, the sampling criteria included athletes from the UK, specialising in the 800, 1500, 5000, 10000 m or marathon disciplines, who had competed for Great Britain (i.e., England, Wales, Scotland) in the final of a global championship in athletics (i.e., Olympic Games, World Athletics Championships, or World Athletics Indoor Championships) during the last three Olympic cycles (2008–2012, 2012–2016, 2016–2020). Purposeful sampling was used to recruit 11 athletes (Male = 6, Female = 5) between 23 and 47 years of age, all from the UK. Athletes were either active (*n* = 8) or recently retired (*n* = 3) and all had undergone blood testing, urine testing, in competition testing and out of competition testing during the course of their careers. Nine of the athletes either had been, or were currently, in a Registered Testing Pool (RTP) and have therefore been subject to regular out of competition testing. None of the participants have ever been sanctioned for a doping offence, but it is accepted that this does not guarantee that they have never used performance enhancing substances or methods.

### Data Collection

The interview guide was developed to address the research questions and refined throughout the data collection process, following reflections after each interview. The interview guide was semi-structured and divided into five main sections. Section one explored participants entry into the sport and their first awareness of doping as an issue (e.g., when you were getting started in athletics, was becoming an elite athlete a goal of yours?). Following this, section two examined the transition into elite sport, the challenges associated with making that step, and the perceptions of doping at that level (e.g., what views and opinions about doping did you notice in your environment?). Section three asked participants about their greatest sporting accomplishments, and about the impact of doping on their careers (e.g., how were you able to achieve your greatest sporting success so far? Did you feel like you were competing on a level playing field?). The fourth section gave participants an opportunity to speak about any crises throughout their careers, and to discuss whether doping had an impact during those low points, and the final section asked participants to reflect back on their careers so far and asked for lessons learned about doping and anti-doping during a life in elite sport.

The average length of the interviews was 76 min, with a range of 45–105 min. The interviews were conducted in person or via video calls, audio-recorded, and transcribed verbatim for data analysis. The interviews were all conducted by the first author who has undergone post-graduate training in qualitative research. Furthermore, as an “insider” in the elite British distance running scene, the first author was able to converse freely with the participants on the nuanced issues of doping within distance running. This promoted free-flowing interviews in which the somewhat sensitive issues being investigated were tackled in a natural and candid way.

### Data Analysis

The data were analysed by a six-step thematic analysis (Braun and Clarke, 2006). Reflexive thematic analysis was specifically selected because of its emphasis on the subjectivity of the researcher, as well as for its concerted engagement with the data during interpretation (Braun and Clarke, 2019, 2020). The six-phases of reflexive thematic analysis were conducted by the first author using NVivo. During phase one the transcripts were read and re-read while listening to the audio-recordings, to promote familiarity with the content. Through this initial phase, notes and points of interest in relation to the research questions were noted down. Phase two then involved inductive initial coding of the sections of data that were pertinent to the research questions (e.g., “normalisation of doping in certain countries makes it more acceptable,” “the possibility of earning large amounts of money as a motivator for doping”). In the third phase of analysis, the coded data were grouped into subthemes (e.g., “variation in doping prevalence by country,” “money as a cause of doping”), and themes (e.g., “doping as a product of certain sporting environments”). In phase four, the themes were viewed as a whole data set to determine whether they formed a coherent pattern and whether all coded extracts related to their themes. Phase five involved reflecting on the themes with the second author and refining the focus of each theme based on those reflections. The second and third authors served the role of “critical friend” (Smith and McGannon, 2018) and encouraged reflexivity by challenging the first authors' construction of knowledge. Each theme was also defined at this stage. Finally, phase six involved selecting appropriate quotes to support each theme and using those to write up of the results section.

### Quality Criteria

In line with our interpretivist philosophical position, a “relativist” approach (Sparkes and Smith, 2009) can be used to judge the quality of the current study. The relativist approach involves formulating a list of characteristics for a particular approach to inquiry, rather than a universal set of criteria which can be used to evaluate any form of qualitative research (Sparkes and Smith, 2009). Therefore, the following criteria act as a starting point for judging the current study: Firstly, as illustrated in the introduction, doping in elite distance running is a relevant, timely and significant issue (i.e., worthiness of the topic). Secondly, rigor was ensured through recruiting information rich participants (i.e., “clean” elite distance runners), who engaged in in-depth interviews, and produced rich data on the impact of doping (and anti-doping) and the reasons for, and barriers to, competing “clean” (see below). In addition, the second and third authors acted as a “critical friends” by providing feedback and reflections on the data throughout the analytical process. Participants also provided feedback on their own interpretations of the data (i.e., member reflections and ethical practice). Thirdly, methodological coherence is demonstrated through clearly interconnecting our philosophical position (i.e., interpretivism), research questions, data collection methods (i.e., semi-structured interviews), and analysis (i.e., reflexive thematic analysis). As a final point, it is important to note that the quality of the analysis can be assessed using Braun and Clarke's (2020) tool for evaluating thematic analysis (i.e., 20 evaluation questions to guide assessment of research quality).

## Results

The participants interviewed in this study have all lived with doping and anti-doping as constant features throughout their careers, and as a result have developed strong views on the use and detection of prohibited performance-enhancing drugs (PEDs) in elite endurance running. Data analysis generated four key themes: Competing clean as a given, anti-doping system viewed as imperfect, doping as a product of certain sporting environments, and doping affects the lives of clean athletes. The results are summarised below in Table 1.

**Table 1:** Summary of results.

**Themes**	**Subthemes**	**Definitions**
Competing clean as a given		Competing clean is so intrinsic to the very reasons that the athletes compete in sport, that it is the only stance they would ever consider.
	Value of the process and experience	Winning is not the only goal for clean athletes, they also place value in the experience and process of being an elite athlete, and therefore take joy and pride from achieving their performances in an honest way.
	Clean identity as a product of the wider sporting culture	A commitment to clean sport is reinforced by the athletes' interpersonal environment. When everyone surrounding an athlete is committed to clean sport, it is easier for the athletes to follow that path.
Anti-Doping system viewed as imperfect		Athletes perceive numerous problems with anti-doping, both from their own experiences with the system, and from their observations of the situation around them.
	Inherent fallibility of anti-doping	There is a strong feeling that certain athletes are still able to get away with doping, and that anti-doping is therefore still unable to detect or prevent all cases of doping.
	Variations in testing protocols between countries	The discrepancies in anti-doping systems in different countries are keenly observed by athletes and are a major source of frustration.
	Suboptimal distribution of tests	There is a feeling that tests are not always deployed in the most effective way—in some cases too close together and in other cases far too infrequently, with little logic to be inferred in either instance.
	Corruption within anti-doping	The corruption scandals that have been uncovered, namely the systematic doping and coverup in Russia and the doping coverup in the IAAF, have frustrated athletes and dented their confidence in the authorities.
	Anti-doping can be difficult for clean athletes	There is a burden that comes with being a regularly tested athlete and there is a feeling that the anti-doping organisations are not always mindful of this in the way they operate and interact with athletes.
Doping as a product of certain sporting environments		A combination of cultural factors may contribute to making the likelihood of doping much more likely within particular sporting sub-cultures.
	Variation in doping prevalence by country	Certain countries are viewed as having more of a problem with doping than others, and the causes of this variation are felt to be cultural.
	Money as a cause of doping	The possibility of earning large sums of money was viewed as a potential motivator for doping, particularly among athletes who come from a less wealthy background.
	Support personnel as a way into doping	The support personnel were identified as a potential route into doping, particularly for more vulnerable athletes.
Doping affects the lives of clean athletes		By pursuing the career of an elite distance runner, the participants have felt the impacts of doping and been negatively affected by it in several ways.
	Financial implications of doping	The career earnings of clean athletes were felt to be severely impacted as a result of the doping of others.
	Emotional implications of doping	The emotional cost on clean athletes who have devoted a lifetimes' work to achieving sporting success, only to be denied by dopers on the biggest stage, is high.
	Implications of others doping on the performances of clean athletes	Continuing to perform at an elite level, while being aware that doping is prevalent at that level, is challenging.

### Competing Clean as a Given

All participants strongly advocated for clean sport and gave impassioned explanations for why they compete clean. The participants repeatedly came back to the value they placed on the process and the experience of being an elite endurance runner, over and above any outcome goals. Participants also highlighted how the environment in which they developed shaped their attitudes towards cheating in sport later in life. It was clear that the intentional use of prohibited substances or methods was never an option that was entertained by the participants–competing as a clean athlete was the only way to be and was therefore not a difficult choice that was extensively deliberated over. In the following sections we explore two subthemes, namely how athletes “value the process and experience” and “clean identity as a product of the wider sporting culture,” in more detail.

#### Value the Process and Experience

The participants' fundamental motivations for competing in sport were interwoven with their commitment to competing clean. Despite the very high level of success that the participants achieved, success in and of itself held little value, and was only meaningful to the athletes if they felt that they had achieved it in a fair way that reflected their own ability and training. The quote below, from participant 2, exemplifies this viewpoint:

“It's not like I thought there needed to be a deterrent … if you'd said to me, you could dope and never be caught and no-one would ever know, it would have no value to me. Yeah, you'd make loads of money, but it would have been a completely pointless exercise in achieving something totally undeserved.” Participant 2

When considering where their “clean sport values” come from, the participants expressed the belief that these were an extension of the values of honesty, integrity, and fair play, which were instilled in the family setting during the participant's childhood. These values created the bedrock for the unwavering commitment to clean sport as they moved through their sporting development.

#### Clean Identity as a Product of the Wider Sporting Culture

Alongside the influence of their immediate families, the participants for the most part also suggested that the broader sporting context in which they developed as athletes, was a “safe” environment, where doping was condemned to such an extent that it was never considered as an option. Participant 3 explains in the quote below how their environment minimises the risk for doping:

“I'm lucky that my coach would never, ever, allow me to come into contact with any of that, so I think I'm in a pretty safe environment away from that sort of stuff. Obviously we are still aware that it goes on but the fact that everybody looks upon it so judgmental from Britain means that you would never consider doing that in the first place.” Participant 3

Throughout their careers, the participants seem to have been able to hold on to the intrinsic value that originally attracted them to the sport. They all wanted to see how good they could be, and how fast they could run, off the back of their own hard work and talent, and so doping never even came into the equation. The participants suggested that their support personnel, and the culture of British endurance running in general, established and reinforced the attitude that sport was only worth doing, if they were doing it clean.

### Anti-Doping System Viewed as Imperfect

From the perspective of the participants, the anti-doping system is currently falling short in several ways. Firstly, there is the clear feeling that certain athletes are still doping and not getting caught. This is compounded by a perceived disparity in the standards of anti-doping in different countries, and further exacerbated by the corruption that has existed at high levels in athletics administration in recent years. Closer to home, it is also felt that testing within the UK is not always optimally distributed. Not only that, but participants in general gave an impression of an anti-doping system that lacks a certain element of human understanding in the way that athletes are dealt with and communicated with. These imperfections in the anti-doping system are explored in more detail in the following five subthemes.

#### Inherently Fallibility of Anti-Doping

The overriding impression from the participants is that there are several ways in which endurance runners can get away with doping without detection. It was felt that the more “sophisticated” methods of doping, such as micro-dosing, were still impossible to detect, and that it was more often the careless or stupid dopers who are being caught. Lack of testing at training camps, and short detection windows were also cited as examples of factors that may enable some dopers to “beat the system,” an example of which is given in the quote below:

“I'd do it when I was away at altitude on camp… your blood would be so different from the altitude… And they couldn't find us all to notify us anyway, because we were scattered all around the complex. You could have easily run away. There were stories from [name]'s place in Kenya where people were jumping walls and stuff when the testers had been coming. It would be so easy to evade.” Participant 3

Participants went on to highlight how certain coaches, athletes, and training groups appeared to be doping without detection, and that this was a further reason for their lack of faith in the system. For example, the case of Somalian coach Jama Aden, who was arrested in a hotel in Spain and found in possession of vials of EPO, was brought up by participant 2–“They didn't catch anyone in Jama Aden's group. They knew they were taking EPO and they found them with EPO and still none of those athletes tested positive.”

Another example from participant 4, where the IAAF (International Association of Athletics federations, now known as World Athletics) essentially admitted the shortcomings of their own anti-doping system, make the imperfections of the anti-doping system all too clear to see:

“I'd emailed the IAAF about it–there was a race after they had come back from a ban, and they both ran [race time]. So I wrote and said, “they ran [race time], that's 4 s faster than her PB and she's just had a ban, what's been going on?”… They caught [Athlete 1] and as soon as they did, they sent me the link to the press release and they said, “we've got [Athlete 1], now for [Athlete 2]”. So I just replied, “that's great to see, and I'm confident that, with the testing procedures in place, it's only a matter of time,” and the email I got back was–“yes, but the Olympics come too soon.” So I was just like… right!! They were saying, “[Athlete 2] is going to run in the Olympics, and we'll catch her at some point.” It was good when they caught her afterwards but it was just… it put a shadow on the whole thing” Participant 4

#### Variations in Testing Protocols Between Countries

The observed differences in the anti-doping procedures in some countries can lead to feelings of unfairness and anxiety around testing. Participant 4 trained with athletes of various different nationalities during their career, and expressed frustration that the doping control procedure was not always the same as the very strict process that was followed for UK athletes:

“We have a Canadian athlete here and she mentioned that the tester had called her to say that she was on her way, and I was like, “she did what? She called her to say she was on her way?” So I contacted UKAD (UK Anti-Doping) about it and I got a response back saying that different national governing bodies operate slightly differently. UKAD are very, very strict in their rules. I couldn't have a phone call or any notification at all. We used to go to France and it was on a gated community, so you would have to ring the buzzer at the gate to connect to the house, and they weren't allowed to do that for some reason. They needed to have the code to get through the gate because otherwise it was a prior notification… I mean when you think of Christine Ohuruogu with the three missed tests, the phone call could have made all the difference to her, and its reputation and things like that which you can't get back. It seems a lot lighter in other countries with other policies, so it's disappointing that there wasn't more IAAF testing and it was all done through UKAD. It would have been nice to know that everyone was under the same level of observation as the British athletes are.” Participant 4

Further procedural differences were observed by the participants in their dealing with various Anti-Doping Organisations (ADOs). Given the fact that athletes' reputations and careers ride on the results of every single doping control, there was some understandable unease at being tested under different conditions while training or competing abroad. Examples of how variations in anti-doping processes can create feeling of vulnerability are given in the quote below:

“I felt really vulnerable by the USADA (United States Anti-Doping Agency) Testing protocol. I asked to see the BCOs (Blood Collection Officer) credentials and he didn't have them. I could have been handing my blood over to anyone… The methodologies across countries differ… I've experienced it all over Europe, the Portuguese protocol is different to the Spanish, which is different to…—the worst one was in India. They took things quite literally. I mean… when you go in with your female chaperone, normally you can drop your pants and kind of squat and get the job done… But in India, they were asking for full frontal 360° views, and you're like “oh my god, that's almost indecent exposure!” People don't talk about that, but I've felt very vulnerable and indecently exposed at certain times, and that's the level that a clean athlete has to go to.” Participant 11

#### Suboptimal Distribution of Tests

There was a strong desire expressed among the participants to see anti-doping done well, but it was sometimes felt that the current testing regime in the UK was not making the best use of the available resources. The participants expressed the feeling that the testing was too predictable, and that the different bodies responsible for anti-doping testing were not doing enough to coordinate their testing programmes. Social media posts from other athletes showing themselves being tested were highlighted as one way in which athletes can often be forewarned that they might be tested later that same day. Another example was provided by Participant 7, in the quote below:

“The testing that we have now, I feel like it can be quite predictable. Like, you run a good race and the drug testers come three times in a week, it's a bit obvious [laughs]… And obviously one of them will be IAAF, one of them with UKAD and then another one might be IAAF again… I think there needs to be better coordination between UKAD and IAAF. I even said to [partner's name] the other day, “Oh, I'm going away next week, I bet the drug testers come” and literally 2 days ago they came, it's just so obvious.” Participant 7

Furthermore, even though the level of athletes in the sample was quite similar across the board—all have at one time competed in a World Championship or Olympic final—there were large discrepancies in the amount that different participants were tested, and this led to the participants feeling either under-tested or over-tested. Participant 5, a recently retired athlete who had a long and distinguished career including winning a medal at a European Championships, fell in the former group and felt that they were not being properly monitored by the anti-doping testing regime, as expressed in the following quote:

“The amount I got tested was ridiculously low. I reckon I got tested four or five times in my whole career… I almost got offended by it to be honest because I was just like “what the ^****^? This is ridiculous that I get tested this little”… the people I used to train with could never believe that I wasn't in the testing pool and they were always like “if it wasn't you, I'd be so suspicious” [laughs]. So yeah, I think there's some major flaws with how people are tested.” Participant 5

However, other participants were tested much more frequently, to the point that it can be quite disruptive, as described by participant 7: “When they come multiple times in a week, then I find it a bit overwhelming, it's just a bit too much.”

#### Corruption Within Anti-Doping

Beyond the concerns that the anti-doping tests were not being optimally distributed in the UK, significant disappointment was expressed at the apparent lack of action from global sports leaders, such as the International Olympic Committee (IOC), to address the sporting cultures within which widespread doping is allowed to perpetuate. Participants' cited the punishment (or lack thereof) following the Russian doping scandal, as an example of this, as in the quote below from Participant 1:

“With a country like Russia, I think the culture is so deep-seated that it could take a generation to change, and even now they're dragging their heels and won't admit that the state was orchestrating the cheating, so you kind of think “well, how is it ever going to change, if even now when it's been proved what they've done, they're not willing to put their hands up and say “okay, we cheated”—it's hard to see how it would change… I'm sort of more frustrated with the IOC, because I think Bach (*Thomas Bach, IOC president*) is just… he's a mate of Putin's, and he looks like he's doing deals behind people's backs. I mean, they had at least two doping failures at the winter Olympics, so obviously they're still doping.” Participant 1

Participants could also perceive unfairness within the system when athletes that they strongly suspected of doping were still being allowed to compete. The frustration felt by participants in those instances was very clear, and that can be sensed in the following quotes:

“They will ban people that are big enough so it seems like they are doing their job, but not big enough to make the sport look like s^***^… To me it is all about image. There's certain athletes that you are like “how on earth, how is [name omitted] still getting away with it?” It just beggars belief.” Participant 10

“I was sixth at the championships, [name omitted] won, [name omitted] was second… they were both definitely cheating. And then the next three I don't know for sure about, but I know for sure about the first two… and I know for sure about countless Algerians and Moroccans that I raced throughout my career who I couldn't even name because there were so many of them… so that's frustrating.” Participant 2

#### Anti-Doping Can Be Difficult for Clean Athletes

Despite their misgivings about anti-doping, the participants were happy to submit to doping controls and took a great deal of care to ensure that their whereabouts were always up-to-date and accurate for out of competition testing. An example of this commitment is highlighted by participant 8, who has recently been added to an RTP, below:

“It's not an inconvenience. I like having to do it, it just means I've reached a certain level of success, so it's cool, it's not a pain. I mean, we travel around quite a lot at certain times of year, so I have to plan that out a little bit. But I have a reminder on my phone every day to double check it, so it's not a big problem, but it is something that needs to be taken very seriously. I don't even want one missed test on my record.” Participant 8

However, over time, participants felt that the anti-doping process can become onerous, stressful, and burdensome. For example, participant 11, who has been on an RTP for over a decade, felt that the level of commitment required of clean athletes was quite extreme and that this is not always appreciated by the anti-doping authorities, as explained in the following quote:

“If I'm at a dinner party at my parent-in-laws and we've had some wine, I might think “oh I don't want to drive home, I'll stay at my parent-in-laws”—then it's “oh, s^***^!!,” waking up in the middle of the night to quickly update your ADAMS (Anti-Doping Administration and Management System) when you remember… or it's Christmas, you go and stay with family, and you're having to think about ADAMS, or you go to a festival, you're like “how do I tell ADAMS where the hell I am at a festival?” But because of my personality type, I've got to tell them exactly where I am. Then checking medications, updating your hotel location, when half the time when you're sent to these races, you have no idea which hotel you're going to be in. Another example, I live in a flat and we share an entrance to our front door and I have to ask the neighbours, “can we keep the bottom door open?” They say, “why, it's cold in the winter, the leaves come in…?”–“I'm really sorry, but I get drug tested and I need them to come straight up to my doorbell, so they know they can get to me.” All these small little things, the effort it takes to be a clean, elite athlete… it's like this subconscious anxiety that I've had for years, not that I'm going to get caught because I haven't done anything wrong, but even though I'm supplying my own blood and urine, there's always this slight doubt of “was there anything in that?” Just from life, because it's so scary. I think it's pretty tough.” Participant 11

Another concern for athletes who are tested frequently, is that the testing process can potentially cause harm. Participant 7 gave a specific example in which a doping control in the UK did not go smoothly and lead to a traumatic experience:

“The guy came to my house to take blood and he asked me which vein they usually use, and I said, “Oh, I usually have this arm done.” But I don't like to look when I have a drugs test, I just look away. He said, “Could you point to the vein that they use?” and I said, “I don't really want to look.” And then he goes, “Can you put the light on?” and the light was on. So my boyfriend brought over a big spotlight that we had in the corner and put it next to us. And then before he even put the needle in, he was saying, “Can you stop moving?” and I said, “I'm not moving.” And then eventually he put the needle in and he literally put it straight through the vein, so blood was spurting everywhere, all over the pillow in my room. So he took it out and I looked down at my arm and I had a massive lump there which I now know is called a haematoma. And then he said, “OK, we have to try again” and I was like “Okay.” And I was obviously crying at this point. So I gave him my arm and he tried it again and he was like, “Stop moving” and I was like, “I'm not moving” and my boyfriend was like, “she's not moving” and again the same thing happened. He was saying, “I can't find your vein” and it was right there. And then he said, “Let's try the other arm,” so he tries the other arm, same thing happens. And then I said, “You're not trying again” and I was crying, my boyfriend was getting mad. So he had to call up someone and say that we tried three times and he couldn't do it.” Participant 7

The participants all acknowledged that it was necessary for a strict process to be followed during anti-doping testing. However, it was also felt that the authorities can sometimes make life more difficult for a clean athlete than it need be, and that the way they deal with athletes lacks a certain human understanding. The communications and methods employed by anti-doping organisations can make clean athletes feel accused or suspected, even when they have done nothing wrong. The observations depict an approach from ADOs towards clean athletes that is adversarial and lacking in open communications. Participants also raised concerns around the timing of anti-doping controls and expressed a degree of consternation at being required to submit to blood testing <6 h before the final of a major championship, and at the anti-social timing of out-of-hours out-of-competition testing.

### Doping as a Product of Certain Sporting Environments

The athletes in this sample are uniquely placed to appraise the prevalence and causes of doping in distance running, having all themselves competed as elite distance runners and lived within that environment for many years. This theme illustrates how the sample view the levels of doping among their competitors and the potential precipitating factors that may lead to doping in this specific event group.

Firstly though, it is important to consider how the participants knowledge on doping within their environment is constructed. Given how impactful the levels of doping are on the ability of these runners to win races, earn money, and achieve their goals, it naturally became an often-discussed topic among athletes, as explained by participant 5:

“I began to look at things in a very different light after running on the professional circuit for a little while, when people talk and you hear stories and you hear rumours. And people come from nowhere and make huge, dramatic improvements and you do obviously question it straight away. So I learned a lot in that period, in terms of how athletes view other athletes… I think there's quite a close-knit group, even if you don't train with some athletes you still become quite good friends with them, and you go to meets and you see who hangs out with each other, and I think it becomes quite obvious from an early stage who is probably clean and who isn't just from the circles that people put themselves in… sitting at dinner, talking to other athletes through friends of mine, and just realising–maybe this is quite a big problem.” Participant 5

The participants developed a view on the issue of doping through their interactions with other athletes, but also through their own understanding of what is physiologically possible as an elite distance runner, and what is not. Participant 3 reiterates below how these impressions are formed and how certain performances and behaviours are viewed as suspicious:

“I think the big one was when [name omitted] came on the scene, for my event, watching that in [competition name omitted], by the end I knew “that's definitely not right” and then you realise, speaking to other people, especially people that have been around these athletes for a long time, you hear stories about… “well when so-and-so was in Font Romeu, they'd only be jogging slowly around the infield” and you start to realise what people are doing and I think the sort of ignorance… that blanket's lifted a bit. So I never directly saw anything that I thought was bad, but I think if you took a survey in the Diamond League, and asked athletes to name who they think in their own event was cheating, I think the same athletes' names would appear. And that probably does mean that they are cheating. [name omitted], who just got done, is one that everyone has always said “like, surely…?” Participant 3

The way athletes' see their sporting environment is profoundly influenced by their competition experiences at the international level. Three specifically influential aspects are explored in the following subthemes–“variation in doping prevalence by country,” “money as a cause of doping,” and “support personnel as a way into doping”–in more detail.

#### Variation in Doping Prevalence by Country

As in the theme above (see section Anti-Doping System Viewed as Imperfect), where it was felt that the way in which the anti-doping rules were being applied varied by country, participants also perceived a difference in the likely prevalence of doping between different countries. This view has built up over time, with the countries which have had numerous distance runners banned for doping being the ones that the participants were most suspicious of. The participants also shared views on what they felt to be the primary causes of doping within distance running and it was again agreed that there would be country-by-country specificities. Extremely different attitudes towards doping were observed depending on the country, and it was generally felt that doping must be viewed differently in the countries in which it has been most prevalent. One of the major factors that can either prevent or promote doping was therefore thought to be the social environment and sporting cultures that exist in a given country, as explained by participant 1, below:

“I've had numerous conversations with [nationality] athletes where I've tried to seek their views on it and they just don't know about it, they don't pay attention, they're just uninterested because for them sport is all about honestly competing against your opponent and respecting the field of play. I think that has a lot to do with how children are educated there, in terms of showing respect to other people, respecting what you do and your possessions, and doing things properly… Then contrasting that with Russia, where I think pretty much all the Russians I've ever competed against must have been cheating, and I think they must believe that what they're doing isn't wrong, because otherwise how would they do it? Some of them must have a conscience. So I think the environment they're in must be telling them “this is what you do in elite sport, the whole world does it, it's perfectly fine,”—“if you win the Olympics, you've still won, you still have to go out there and run the race.” Participant 1

#### Money as a Cause of Doping

Almost all participants made the observation of money as a potential motivator for doping, and again noted that their feelings towards clean sport are a product of their environment and that attitudes would therefore not be the same across all environments. The following quote highlights this:

“It's all very well me sitting here saying these things from the perspective of a white, privileged person but if I'd grown up in poverty, somewhere with no food, no money, and running is a way out for me, it's not just a case of… “oh yes I'm going to be the best I can be today!” It's like “I'm going to feed my family for the next 5 years…,” so it is a really difficult one…” Participant 4

The participants made particular reference to Kenya and the possible causes of their doping cases. Money was again felt to be a key factor, as described the following quote:

“For some people, it's a no-brainer. The amount of money you can get and the consequences that you get from these situations is crazy… Like some of the Kenyans, they're not in the sport to have a great time; they're trying to get some cash and they can get cash by getting to a world championship final and being top five. And then you know, yeah they get caught and they're out for 2 years, but they don't get fined. They get to keep all this money.” Participant 8

However, not all participants felt that the need to earn money was alone enough to explain why a distance runner would turn to doping. Participant 9 expressed the opinion that there must be a tipping point in terms of attitudes from an athlete getting started in sport, to that same athlete later in their career deciding to take PEDs:

“No eight-year-old kid that starts sport thinks “this is how I'm going to become a millionaire…” so, I think something has to change somewhere on the way where some people sort of abandon their original goals and are then willing to cheat and try and make money.”

#### Support Personnel as a Way Into Doping

According to the participants, one way in which an athlete's attitude might change from when they first enter the sport, to when they make the decision to use PEDs, is through the influence of the athlete support personnel. The participants consistently identified the athlete support personnel as a potential route into doping, especially given the vulnerability and/or naivety that is common among young athletes, and athletes who are at a low ebb in their careers. Participant 2 presents how this path to doping could open up in the quote below:

“It only takes one person to say “well, what if you tried this?” or “have you spoken to this guy?” or “my mate did this,” and all of a sudden that door has been opened to something that you shouldn't even ever consider. But athletics is a lonely sport, sport is lonely, so it's really easy to imagine a situation where someone's at a real low, like I was at various points, and someone you do trust, or you've come to trust through making bad decisions, just whispers something, like a throwaway comment, whatever it is, and that can set you on the wrong path.” Participant 2

The agents (otherwise known as “athlete representatives” or “ARs”) were singled out by some participants as members of the athlete support personnel who might be particularly responsible for facilitating, encouraging, or orchestrating the doping of distance runners. It was generally acknowledged that athletes probably would not organise their own doping alone, and it was speculated that agents may therefore be the ones who enable the doping, and the quote below provides an example of that view:

“I don't think athletes are smart enough to organise a doping program. Agents are the ones who will have this pathway created, they have their network, and they can bring in an athlete, and just chuck them out the other side, like a conveyor belt. They have their go-tos. It must be like a mafia thing, and the less the athlete knows, the less the athlete can grass up.” Participant 10

The overall impression is that a combination of the opportunity to win large amounts of money, together with unscrupulous support personnel providing logistical support to aid doping, has created the current situation in which numerous distance runners from certain countries have been banned for doping offences.

### Doping Affects the Lives of Clean Athletes

This theme is more introspective and presents the athletes' considerations of the impact that the doping of other athletes has had on their lives, careers, and livelihoods. It was acknowledged by every single participant in this study that their careers and lives would have been different if there were no doping in elite endurance running. Through the following three subthemes, we now explore how athletes are impacted financially and emotionally by the doping of others, and the potential performance-related implications of competing in an environment in which doping occurs.

#### Financial Implications of Doping

The implication that participants could most easily identify was the financial impact of doping. The loss of earnings at the hands of rivals who are doping can be significant. That stems not just from being bumped down one place in the prize money stakes, but also from knock on effects on contracts and future opportunities, as explained below:

“Doping will have 100% cost me significant six figure sums of money. One example; there's no way [athlete] is clean, and I was the first person to miss out on the Olympic final finishing behind them. If I had made that final, I'd have been a consecutive double Olympic finalist, there's not many of those, and you re-sign your contracts after Olympics. I was on a worse contract than I would have been had I been in the Olympic final… And Olympic finalists often get paid appearance fees, and I obviously wasn't getting any of those either.” Participant 2

#### Emotional Implications of Doping

This impact on earnings was felt by a number of participants, but it was not the money they lost out on to dopers that they regretted most, but the moments that they never got to experience. This is the emotional impact of doping on clean athletes. The following quote takes on particular significance when it is considered that Participant 4 narrowly missed out on winning a global medal, behind an athlete who later went on to serve a doping ban:

“It made a massive influence on a lot of things for me… I mean, financially is one of them, but it's never really been about money for me. It's been about achievement and motivation and that lifelong dream of having a medal. Just that sense of achievement and that moment, I just wanted to know what that felt like to cross that line and to have achieved that. I mean that would have been my whole life's work, everything I could have ever wanted, just that second crossing that line…” Participant 4

The way in which the recently retired athletes reflected back on their own careers was also influenced by the subsequent realisation, or suspicion, that doping may have had a bigger impact than they thought at the time. Some, like participant 1, have been left wondering whether it had all been worth it:

“Since I've retired and all the doping scandals have come to light there have been times where I think “why did I spend 10 years of my life doing that? What does it mean?” Like finishing [position] at the Olympics, when I finished that race, I was slightly disappointed that I wasn't in the medals, but I thought “well, [position] is still a big achievement, I can walk away with my head held high.” Now I'm thinking, I should be bumped up into the medals, but it's never going to happen. I'm left thinking “well, where did I finish?” There's this question mark over it which I don't think—it's never going to be resolved, so I just have to try and forget about it. It's left a bit of a sour taste. Previously I was so proud of everything, but now I'm kind of thinking would I have been better off just sticking with a proper job? What was it all for?” Participant 1

#### Implications of Others Doping on the Performances of Clean Athletes

The way in which an athlete's perceptions of the levels of doping in their event can affect their own mental preparation and performance was also discussed at length by the participants. Currently active athletes tended to be less willing to acknowledge that doping might be having an influence in the results of their races. They accepted that they could not know for certain what their rivals might be doing, and felt it was important not to let suspicions of doping affect their own performance, as in the following quote:

“Do I think there's drugs in sport? Yes, and I think there is a lot of it. But when I stand on the start line, if I think someone next to me is taking drugs, I shouldn't be on that start line. It would be pointless because you're giving them a mental advantage. So you have to train and race like it's a clean sport, because that is the only way you can get the best out of yourself.” Participant 10

Opinions were split among the participants on whether it was possible to be the best distance runner in the world as a clean athlete. Participant 8 had recently made the jump up to becoming one of the very best in the world and felt that doping had become less of an issue for them, because they now believed it was possible to compete with anyone in the world, regardless of whether they might be doping, as described below:

“This year is probably the first year where I'm like, “Okay, I can beat them without doing anything.” For me, it was “we work hard, we work hard, we work hard, we see how good we get,” but in my head, it was difficult to see myself being the best in the world, especially if we're going up against people that are getting these extra percentages and stuff. But now I won't even think about these guys who are doping because I'm going to beat them anyway.” Participant 8

Conversely, participant 4 explains below how they tried to ignore the likely doping of their competitors, so that they wouldn't be at a psychological disadvantage, but that it was not always possible do so:

“Deep down, I knew I couldn't win. But you try and tell yourself that you can. I was through to the final so anything can happen in the final. But I just knew that if it was a fast race I couldn't match them, and if it was a slow race I was going to be wanting… In the end, I was on the start line and I didn't think about that, I was saying to myself “I'm just going to do what I can do.” So you try to be positive but I just knew I couldn't match them and that was hard, because I didn't feel like I had a chance. That's what really upset me about it… I almost wish I didn't know, because then at least I'd have been a bit more naïve going into it.” Participant 4

Perceptions on whether it was possible to reach the very top as a clean athlete also depended on the specific event being considered. The women's 10000 m and marathon events were highlighted as events where winning clean in recent years appears to have been almost impossible, whereas Emma Coburn, World Champion in the 2017 3000 m steeplechase, was provided as an example of a clean athlete winning a global title.

For those athletes who have concluded that doping is prevalent at the top of their event, that must inevitably affect their perception of what is possible for themselves as clean distance runners. Participant 9 said “I'm not necessarily pessimistic, but I just think you have to be real,” and followed up with the following assessment of the current situation:

“I was asked recently–“do you think you can win an Olympic medal clean?” And I said, “I don't think you can.” That's such a horrible, and such a sad, thing to say, because at the end of the day that's what a lot of us are striving for. But honestly, for me, I don't think you can at the moment.” Participant 9

Having results skewed by doping can also lead to loss of confidence in training programs, decreased motivation, and impaired decision making to try and keep up with the unattainable levels achieved by PED users. Participant 6 explains how this scenario played out in their career:

“We were killing ourselves trying to keep up, as clean athletes… and we didn't even realise because we just wanted to race and do the best we could. It didn't even occur to me, like, “Oh my gosh! My body physically can't do that without drugs.” I was just trying to run the best I could. But I felt a lot of anger for a long time when I was injured. I've spent a lot of years injured. My body was almost in recovery from battering it so much but not really realising that I was overtraining. I think the main reason I've been side-lined has been overtraining and fatigue. That can only really come from just pushing yourself so much. It just angered me.” Participant 6

One quote that summarises this theme comes from participant 11, who when asked whether they felt their career had been affected by the issue of doping, replied “I would say immensely. Immensely disrupted… I think my career has probably been impacted dramatically in terms of positions, success, mental health, monetary values.”

## Discussion

The aim of this study was to better understand athletes' reasons for competing clean, their perceptions and experiences of the anti-doping system, and the impact of doping in their environment on their lives. Reflexive thematic analysis of the interview data generated four key themes: Competing clean as a given, anti-doping system viewed as imperfect, doping as a product of certain sporting environments, and doping affects the lives of clean athletes.

### Doping Prevention Measures Do Not Acknowledge and Accommodate for Athletes Committed to Clean Sport

The participants' motivations for being an athlete were inexorably linked to doing it in an honest and fair way. The athletes presented a high level of intrinsic motivation, with a desire to test their natural limits being a primary driving force. The participants' commitment to clean sport appears to derive from the values and morals they were raised with, and this finding is consistent with previous research (MacNamara and Collins, 2014; Erickson et al., 2015; Petroczi et al., 2021). Another factor contributing to participants' clean sport attitudes appears to be the sporting environment in the UK, which they felt strongly discouraged doping. This is again in agreement with previous studies which have focused on attitudes towards doping specifically within the UK (Bloodworth and McNamee, 2010).

Recent research (Petroczi et al., 2021) has highlighted what should perhaps have already been obvious—that a significant proportion of athletes are not tempted to use prohibited PEDs. These athletes are not well-served by the current doping prevention measures and this study further supports strengthens the case for changes in anti-doping strategy. The current approach to doping prevention works from a starting point that all athletes might consider doping, but the present research strongly indicates that this is a false assumption. Current anti-doping education provisions, which tend to focus on why it is morally wrong to use prohibited performance-enhancing substances and/or methods, and the sanctions and health risks associated with doing so, will have no impact on preventing doping among established senior athletes who have already committed to clean sport. There has not been enough thought given to how anti-doping can best serve the significant group of athletes who would never knowingly use prohibited substances, but who still face challenges in this area, such as the legitimate fear of their reputation being permanently tarnished by an unintentional anti-doping rule violation (ADRV). Further research is required to determine how anti-doping can meet the needs of all athletes.

### Globally Effective Anti-Doping Is Necessary to Win the Trust and Support of Clean Athletes

The anti-doping system is viewed as an essential component of a fair and credible sport, but it was also felt that there are aspects of the current approach which are not best optimised, both at home and abroad. The belief was that doping is still going undetected among international level distance runners, particularly among athletes from certain nations. Coupled with the highly publicised reports of corruption of the anti-doping process at the very highest levels within athletics (Ingle, 2020), it is easy to understand why participants' confidence in the anti-doping system was low. This is concerning, given that perceptions of anti-doping legitimacy are related to how athletes feel about the anti-doping rules (Woolway et al., 2020).

Difficulties with testing athletes out of competition in remote locations was another of the issues raised. Participant 3's observation that athletes in Kenya may have been attempting to avoid testing by “jumping walls” proved to be foretelling, as a Kenyan distance runner has since been banned for 3.5 years for evading an out of competition test, and it was noted in the decision that “an independent witness confirmed that he had seen the athlete hurdling over a fence to escape from the compound” (Athletics Integrity Unit, 2020). Concerns were expressed not only at the difference in perceived standards of anti-doping testing from country to country, but also at the variations within the testing procedures. For example, some ADOs will provide athletes with prior notification for an out of competition test, via a telephone call, whereas the British athletes in this study were always required by UKAD to go to every length to ensure that they could be tested with no prior notification whatsoever. This issue was brought into the spotlight when the 100 m world champion, Christian Coleman, was sanctioned for 2 years for whereabouts violations. Coleman's case hinged on the fact that the AIU doping control officers (DCOs) did not telephone him to alert him that they were trying to locate him for testing, as had previously been the case when he was tested by USADA (Sport Resolutions, 2020). This highlights the absolute need for consistency between ADOs to make it as easy as possible for athletes to comply with the rules, and also to give athletes the peace of mind to know that they are being treated fairly and equitably.

The impact of doping on the international stage on the lives of clean athletes cannot be underestimated. Most of us can recognise and relate to the joy of sporting victory, and the agony of defeat, whether that be as a spectator or a competitor. But just as we all move on from our experiences of sporting highs and lows, so too the elite athletes in this sample took a philosophical outlook and accepted being beaten fairly by better athletes. However, what was harder to accept were the moments that they had been cheated out of. A fair loss is one that can be dealt with. Losing as a result of cheating is much harder to swallow. For example, listening to Sir Bobby Robson at the end of his long and illustrious career in football, you can hear how much the “Hand of God” goal, scored using the arm by Diego Maradona, was still hurting him (BBC Radio 4, 2004). Unfortunately, you get the sense that the losses at the hands of doped athletes experienced by some participants in this study may be equally hard to accept.

World class sport must be supported by world class anti-doping. In recent years, as shown by the Russian scandal and the IAAF scandal, global anti-doping has actively been working against clean athletes. But even setting aside those examples of corruption, anti-doping must do more to convince clean athletes that no stone is being left unturned in the defence of clean sport. Communication from ADOs to athletes is also essential to demonstrate the lengths that the ADOs are going to protect the interests of clean sport. Clean athletes can be the strongest supporters and advocates for the anti-doping movement, but only if they have trust and belief in the systems in place.

### The Decision of Which Athletes to Include in an RTP Must Not Be Taken Lightly

The data also revealed a difference in the required commitment to anti-doping between athletes included in an RTP, and those who are not in such a pool. RTP athletes have a responsibility to provide accurate whereabouts information to ensure their availability for no notice out of competition testing for 1 h of every day of the year and this necessitated a significant investment of time and emotional energy, given the extremely damaging consequences of a whereabouts violation. Two previous surveys of Dutch and Danish athletes showed that a third to four tenths of respondents felt that the whereabouts system has a negative influence on the pleasure they experience in being an elite athlete (Overbye and Wagner, 2014; Valkenburg et al., 2014). For a few athletes in this sample, anti-doping and the associated obligations were a source of stress, due to the risk of a positive test from a contamination, fear of a whereabouts failure, unpleasant experiences during doping controls, or lack of understanding from the ADOs.

Given the significant imposition that results from inclusion in an RTP, one would hope that decisions on which athletes to include in the RTP were carefully considered and based on solid evidence of doping risk. However, there is very little logic to be inferred when looking at which athletes in this sample have been included in the RTP, and which have not. The only discernible difference between the two groups is that participants who were identified by UK Athletics (UKA) and placed on the talent ID pathway early on in their careers appear to have been added to the RTP at the same time. The other participants, who developed later or through a different pathway, such as the US Collegiate system, were rarely added to the RTP and were subsequently tested much less frequently over the course of their careers, despite ultimately reaching a very similar performance level.

In a recent statement, UKAD's head of testing said: “Our testing continues to be risk-based and intelligence-led. Every test we do, there is a huge amount of time and effort that goes into that to make sure we are testing the right people, at the right time, for the right substances” (BBC, 2020). It would be interesting to understand how UKAD justify the large and seemingly random discrepancies in the frequency of testing experienced by participants in this study. As for the participants themselves, they viewed the highly variable testing levels as evidence of a system which is not achieving the best coverage of the population from the number of tests available.

### Clean Athletes Can Recognise Causes or Potential Indicators of Doping

Another key aspect emerging from this study is that elite athletes are highly attuned to doping in their specific events and are perhaps better placed than anyone else to identify the early indications of doping. This insight derives from a detailed understanding of what is physiologically possible, in terms of absolute performance levels, performance progression, and the repeatability of high-level performances. Naturally, athletes also take a keen interest in the training of their peers and rivals, and this has included observations of how blood doping appears to minimise the need for endurance training and facilitate more and higher intensity speed training, without the normal deterioration in aerobic capacity. Furthermore, the way that athletes who are doping interact socially also appears to be very different to the social interactions between clean athletes. This level of observation is perhaps only possible for someone who is fully embedded in the life of an elite athlete. If ADOs could better facilitate an open dialogue between themselves and clean athletes, they may be able to gain a deeper understanding of the sport-specific landscapes of doping. Both parties would stand to benefit from such an arrangement. One hurdle to overcome is that clean athletes often will not feel justified in speaking up when they “only” have a suspicion of doping and no hard evidence, so the ADOs must be proactive in initiating communication.

The participants' assessment that the prevalence of doping varies markedly by country is certainly consistent with a recently published paper, which used the haematological parameters from 3683 blood samples collected at the 2011 and 2013 World Athletics Championships to estimate the prevalence of doping among elite distance runners. The study estimated that the prevalence varied from 0% to a maximum of 89%, depending on the country (Faiss et al., 2020), and therefore gives credence to the view that doping is on the one hand being enabled in some countries, whilst on the other hand being strongly condemned and suppressed in other countries.

Unsurprisingly, given the number of elite distance runners from Kenya who have been charged with doping violations in recent years (66 Kenyan distance runners are currently serving doping suspensions) (Athletics Integrity Unit, 2021), the participants had developed viewpoints on what might be behind that issue. One contributing factor was felt to be the goal of some Kenyan athletes to earn money through athletics. Certainly, the motivations of elite Kenyan athletes differ markedly from those of the athletes in this sample, with a number of studies showing that economic success is a primary motivating factor for the elite Kenyan runners (Onywera et al., 2006; Elbe et al., 2010; Wilber and Pitsiladis, 2012). Whether that difference in motivating factors is actually related to an increased doping risk is yet to be fully elucidated.

There are consequences of doping for the athlete who is caught doping, and these have been explored in depth both in the academic literature (Kirby et al., 2011; Georgiadis and Papazoglou, 2014), and through numerous autobiographies (Chambers, 2010; Millar, 2011; Hamilton and Coyle, 2013; Dekker, 2017). However, there are also consequences of doping for the clean athletes who compete against those who are doping, and these have been researched much less frequently. One 2016 study (Erickson et al., 2016) made a start on storying the wider impacts of doping, and this study builds on that work by highlighting the impact that doping can have on the earnings, emotional wellbeing, and performance of clean athletes. This provides a stark reminder, if one were needed, of the critical importance of anti-doping work in the lives of clean athletes.

## Limitations and Directions for Future Research

The reasons and motivations underpinning an athlete's decision to complete clean is highly individualistic and influenced to a large degree by the specific sporting and cultural context within which they compete. The present study provides an insight into why elite British long-distance runners may opt to compete clean, but it is important to note that the findings cannot be generalised to long-distance runners from other countries with different sporting countries, or to athletes competing in different events within athletics, or in different sports entirely. Given that there have been very few cases of an elite British distance runner testing positive for PED, it would be interesting to explore the attitudes towards clean sport among a group of distance runners from a country where cases of doping have been more prevalent.

The existing literature, along with the present study, suggest that the athlete support personnel may play a role in either enabling or preventing doping. Within distance running, the agents have been identified as an influential group who coordinate many aspects of an elite distance runners' career. It would be beneficial to understand how agents perceive their role in the prevention of doping and their views on potential risk factors. Many agents of top long-distance runners have worked in the sport for a long time and may be able to provide a different perspective to the athletes on this issue, with perhaps a higher-level understanding of the economic and systemic drivers of doping.

A further limitation of this study is that the age range of participants is fairly wide, ranging from 23 to 47 years old, and that the sample includes both active and retired athletes. It is perhaps unreasonable to expect that athletes with such varying degrees of experience would view the issues of doping and anti-doping in similar ways. In section Implications of Others Doping on the Performances of Clean Athletes, there was some indication that the views were split between current and former athletes on the influence of doping on their results. Further research is warranted to explore this phenomenon with two separate participant groups, one group of active athletes, and one group who have recently retired. It may however be found that clean athletes who are currently competing do not want to confront or dwell on the issue of doping in their own events because of the potential detriment to their own performance. This could mean that researching this topic exclusively with actively competing athletes could be challenging. On the other hand, retrospective reflection can bring depth and richness to the data but could lack being current and relevant in the fast-changing landscape of elite sport and performance-enhancement.

The current study did not examine gender differences in attitudes towards doping and anti-doping. It would be interesting to explore whether males or females are more opinionated on these issues and also whether males or females had their careers affected to a greater degree by doping. Another potential area for further research would be to investigate whether there is any connection between differences in testing procedures in various countries and the prevalence of doping among athletes in those countries.

## Conclusion

The main reason for having an anti-doping system is to minimise the impact of doping on sporting outcomes and to give clean athletes a chance at success. However, currently, clean athletes suffer negative consequences from both doping and anti-doping. Doping at the international level is the key issue for elite distance runners from the UK. On the UKAD website, they describe themselves as “an active participant in the global fight against doping in sport.” To best serve British athletes, UKAD must follow through on that statement and demonstrably prioritise international collaboration, to work towards a more level playing field across all nations. The global approach to anti-doping must attack the task of doping prevention with passion, skill, and persistence, while concurrently not impeding the lives of clean athletes to a greater extent than is necessary to achieve that goal.

## Data Availability Statement

The datasets presented in this article are not readily available because due to the need to ensure anonymity, raw interview data are restricted to the research team. Requests for granting access for re-analysis of the data, collaboratively or otherwise, will be considered on a case-by-case basis. Requests to access the datasets should be directed to a.petroczi@kingston.ac.uk.

## Ethics Statement

The studies involving human participants were reviewed and approved by Faculty Research Ethics Committee Faculty of Science, Engineering and Computing Kingston University. Written informed consent for participation was not required for this study in accordance with the national legislation and the institutional requirements.

## Author Contributions

JS and AP conceptualized and designed the study. JS facilitated contact with the athletes, collected the data, and wrote the manuscript. JS and ST analysed and interpreted the data. AP and ST contributed to the writing and editing of the manuscript. The final version of the manuscript was approved by all authors.

## Conflict of Interest

The authors declare that the research was conducted in the absence of any commercial or financial relationships that could be construed as a potential conflict of interest.
